# Patients With Chronic Low Back Pain Have an Individual Movement Signature: A Comparison of Angular Amplitude, Angular Velocity and Muscle Activity Across Multiple Functional Tasks

**DOI:** 10.3389/fbioe.2021.767974

**Published:** 2021-11-15

**Authors:** Guillaume Christe, Camille Aussems, Brigitte M. Jolles, Julien Favre

**Affiliations:** ^1^ Department of Physiotherapy, HESAV School of Health Sciences, HES-SO University of Applied Sciences and Arts Western Switzerland, Lausanne, Switzerland; ^2^ Swiss BioMotion Lab, Department of Musculoskeletal Medicine, Lausanne University Hospital and University of Lausanne, Lausanne, Switzerland; ^3^ Institute of Microengineering, Ecole Polytechnique Fédérale de Lausanne, Lausanne, Switzerland

**Keywords:** low back pain, motion analysis, lumbar, kinematics, electromyography, angle, angular velocity, muscle activity

## Abstract

Despite a large body of evidence demonstrating spinal movement alterations in individuals with chronic low back pain (CLBP), there is still a lack of understanding of the role of spinal movement behavior on LBP symptoms development or recovery. One reason for this may be that spinal movement has been studied during various functional tasks without knowing if the tasks are interchangeable, limiting data consolidation steps. The first objective of this cross-sectional study was to analyze the influence of the functional tasks on the information carried by spinal movement measures. To this end, we first analyzed the relationships in spinal movement between various functional tasks in patients with CLBP using Pearson correlations. Second, we compared the performance of spinal movement measures to differentiate patients with CLBP from asymptomatic controls among tasks. The second objective of the study was to develop task-independent measures of spinal movement and determine the construct validity of the approach. Five functional tasks primarily involving sagittal-plane movement were recorded for 52 patients with CLBP and 20 asymptomatic controls. Twelve measures were used to describe the sagittal-plane angular amplitude and velocity at the lower and upper lumbar spine as well as the activity of the erector spinae. Correlations between tasks were statistically significant in 91 out of 99 cases (0.31 ≤ r ≤ 0.96, all *p* < 0.05). The area under the curve (AUC) to differentiate groups did not differ substantially between tasks in most of the comparisons (82% had a difference in AUC of ≤0.1). The task-independent measures of spinal movement demonstrated equivalent or higher performance to differentiate groups than functional tasks alone. In conclusion, these findings support the existence of an individual spinal movement signature in patients with CLBP, and a limited influence of the tasks on the information carried by the movement measures, at least for the twelve common sagittal-plane measures analysed in this study. Therefore, this work brought critical insight for the interpretation of data in literature reporting differing tasks and for the design of future studies. The results also supported the construct validity of task-independent measures of spinal movement and encouraged its consideration in the future.

## Introduction

Alterations in spinal movement have been suggested as one of the key physical factors in the persistence of chronic low back pain (CLBP) (Marras et al., 1995; O’Sullivan, 2005; Dubois et al., 2014), however, the understanding of spinal movement behavior in CLBP remains limited. The abundance of measures used to describe spinal movement, as outlined by two recent systematic reviews (Papi et al., 2018; Moissenet et al., 2021), is undoubtedly one of the reasons limiting a better understanding. Consequently, to advance the field, there is a need to determine if that many measures are needed or if it would be possible to focus on a selection of measures. While characterizing the movement in terms of angular amplitude, angular velocity and muscle activity appears appropriate for a comprehensive description (Laird et al., 2014; Papi et al., 2018; Moissenet et al., 2021), the necessity to test multiple functional tasks remains to be determined.

So far, CLBP spinal movement has been assessed during a range of different functional tasks (Shum et al., 2007; Christe et al., 2016b, 2020; Lima et al., 2018; Matheve et al., 2019). However, these prior works mainly assessed one task at a time, or when multiple tasks were assessed, the influence of the task on spinal movement was not analyzed (Papi et al., 2018; Moissenet et al., 2021). Yet, individual consistency in spinal angular amplitudes across different functional tasks has been demonstrated in pain-free participants (Alqhtani et al., 2015; Seerden et al., 2019), suggesting that it may also be the case for individuals with CLBP and for angular velocity and muscle activity measures. This possibility is particularly supported in the clinical setting, where consistent movement patterns are frequently observed in CLBP patients. If measures from different tasks were to carry similar information (for example, patient X moves with relatively lower flexion than the other individuals independently of the tasks analyzed), this would suggest the existence of “individual spinal movement signatures.” Clarifying this point appears essential, as it would help the interpretation of data in literature and the design of new experiments. In fact, on one side, knowing the extend of movement signatures would provide a basis for the comparison of studies testing different tasks and, on the other side, it would provide a rationale for the number and specificity of the tasks to include in future studies.

If individual spinal movement signatures were to exist in CLBP patients, this would question the possibility to develop task–independent measures of spinal movement. Such task–independent measures could produce more robust assessments and reduce the number of variables to deal with in statistical analyses, which would be beneficial for both the design and the interpretation of future studies. If possible, this simplified description of spinal movement could prove particularly useful to detangle the role of spinal movement alterations in CLBP development or recovery and inform rehabilitation principles (Wernli et al., 2020b; Schmid et al., 2021).

Therefore, the first objective of the study was to determine the influence of the functional tasks on the information carried by spinal movement measures. To this end, this study aimed: 1) to analyze the correlations among various functional tasks in patients with CLBP; 2) to compare the performance to differentiate patients with CLBP from asymptomatic controls among functional tasks. Based on prior research, measures corresponding to peaks and ranges of sagittal-plane lumbar angular amplitude and angular velocity, as well as maximal erector spinae activity were analyzed (Shum et al., 2005; Dankaerts et al., 2009; Hidalgo et al., 2012; Christe et al., 2016b, 2020). Following previous work in asymptomatic people and a pilot study in patients with CLBP (Alqhtani et al., 2015; Christe et al., 2016a; Seerden et al., 2019), it was hypothesized that the spinal movement measures would be positively correlated among the functional tasks. It was also hypothesized that there would be no relevant performance difference among functional tasks (Dankaerts et al., 2009; Laird et al., 2014; Christe et al., 2016b, 2020; Moissenet et al., 2021);

The second objective of the study was to determine the construct validity of task–independent measures of spinal movement obtained by grouping measures across multiple tasks. Specifically, we aimed: 3) to compare the performance of task–independent measures to differentiate patients with CLBP from asymptomatic controls to the performance of task-specific measures. It was hypothesized that the performance of task–independent measures would not be inferior compared to the performance of task-specific measures.

## Materials and Methods

### Design

This cross-sectional case-controlled study is reported according to the STROBE (Strengthening the Reporting of Observational Studies in Epidemiology) criteria (von Elm et al., 2007).

### Participants and Setting

Recruitment took place in an interdisciplinary rehabilitation program (IRP) at the local university hospital. This IRP is a full time 3-week program that includes patients with difficulties to maintain their leisure and professional activity because of CLBP. Participants to the IRP were invited to take part in the study if they met the inclusion/exclusion criteria. Both males and females could participate if they had a diagnosis of non-specific LBP with or without leg pain for more than 3 months, a sufficient French level and an age from 18 to 65 years old. Exclusion criteria for the CLBP group in this study were the presence of a diagnosis of specific LBP and/or previous back surgery that limited spinal mobility (i.e., spinal fusion). Asymptomatic controls were a convenience sample recruited via emails and flyers. To be included, they had to have no history of LBP requiring third-party attention during the last 2 years. They were also excluded in the presence of any recent or current episode of LBP. Exclusion criteria for both groups included pregnancy, a body mass index (BMI) above 32 kg/m^2^ and other concomitant pain or condition that could compromise the evaluation of lumbar kinematics. The BMI cutoff was selected to limit the influence of body shape on lumbar kinematics and experimental complications, without compromising external validity and patients’ recruitment. The research was approved by the local Research Ethics Committee (CER-VD 2018-00188) and all participants signed an informed consent form before enrolment in the study.

### Experimental Procedures

Participants were invited to the movement analysis laboratory at the university hospital for a measurement session before the IRP. First, participants completed three reliable and valid questionnaires to document mean pain intensity during the last 24 h, kinesiophobia and catastrophizing using the numeric pain rating scale (24h-NPRS), the Tampa Scale of Kinesiophobia (TSK) and the Pain Catastrophizing Scale (PCS), respectively (Sullivan, 1995; Vlaeyen et al., 1995; Chapman et al., 2011). Then, after having cleaned the skin with alcohol and shaved it, if necessary, two pairs of electrodes were placed bilaterally parallel to the erector spinae fibers 3 cm lateral to the L3 spinous process (Dupeyron et al., 2013; Wong et al., 2016). Participants then performed one submaximal voluntary contraction in crook lying as described by Dankaerts et al. (2004). Reflective markers were then attached to the participants lumbar region and pelvis following a previously described protocol (Seay et al., 2008; Christe et al., 2016b, 2017, 2020). Lumbar markers were placed on the spinous processes of L1, L3 and L5 with four additional markers attached between these markers on each side of the spine, at a distance of 5 cm. Pelvis markers were placed on the posterior superior iliac spines, anterior superior iliac spines and iliac crest tips. Marker trajectories and lumbar muscle activity were measured using an optoelectronic motion capture system with 14 cameras (Vicon, Oxford Metrics, Oxford, United Kingdom) and an electromyography system (Myon, Schwarzenberg, CH) recording synchronously at 120 and 1200 Hz, respectively.

Data collection started with the recording of a reference standing posture, where participants were standing upright and looking forward with arms at 60° of shoulder abduction. Then, five functional tasks were recorded in the same order for every participant to avoid varying remnant effects among participants as some were more exacerbating for participants: standing flexion, sit-to-stand, stepping-up on a 36 cm high step, picking-up a sponge from the floor and lifting a 4.5 kg box from the floor. All functional tasks were first demonstrated in a video with standardized instructions (Supplementary Appendix SA). Each functional activity was practiced between one and three times and then recorded three times, except for picking-up that was recorded ten times (for the purpose of another study, Christe et al., 2019). Following the video instruction, and before performing each task, participants rated on a zero to ten scale how much do they think the task to-be-performed is harmful for the back (perceived harm; 0: not harmful at all; 10: extremely harmful). After each task, they also rated their pain during the task with a numeric pain rating scale. At the end of the session, participants completed the French version of the Oswestry Disability Index (ODI) (Fairbank and Pynsent, 2000; Vogler et al., 2008).

### Data Processing

Spinal kinematics were calculated based on a three-segment biomechanical model that includes the pelvis and the lower lumbar and upper lumbar spine (Christe et al., 2016b, 2017, 2020). Briefly, markers’ trajectories were used to calculate the orientation of anatomical frames embedded in each segment. The joint coordinate system (Grood and Suntay, 1983) was then used to calculate sagittal-plane joint angles at the lower lumbar (LLSa) and the upper lumbar (ULSa) joints. LLSa was defined as the angle between the lower lumbar segment (L3-L5 central and lateral markers) and the pelvis segment, while ULSa was defined as the angle between the upper lumbar segment (L1-L3 central and lateral markers) and the lower lumbar segment. Angles were low-pass filtered using a 15 Hz Butterworth filter. The amplitude of the angles during the reference standing posture were subtracted from the angle curves to limit the inter-individual variations in morphology. Angular velocity curves (LLSv and ULSv) were obtained by numerical differentiation of the angle curves.

Electromyography recordings were band-pass filtered using a Butterworth filter with cutoff frequencies at 20 and 450 Hz. Then, for both muscles, the minimal amplitude of the electromyography signals recorded during the entire session was identified and this minimal amplitude was subtracted from the signals. This operation defined a zero-value (0%) for the electromyography data. Next, for both muscles, the signals were scaled in order to have the amplitude recorded during the submaximal voluntary contraction in crook lying equal 100%. Submaximal contraction was chosen for the normalization because its reliability was shown to be superior to maximal contraction in CLBP patients (Dankaerts et al., 2004).

In order to extract the movement measures from the angular amplitude, angular velocity and muscle activity curves, first, the curves were time-normalized to 0–100% for each repetition of each task. The beginning and the end of the task were determined visually using strict criteria based on markers displacements (Christe et al., 2016b, 2020). Then, following the methodology in prior studies (Christe et al., 2016b, 2017, 2020), the curves were tested using the coefficient of multiple correlation (CMC) (Kadaba et al., 1989) and the curves presenting a characteristic pattern were described by discrete measures. In total, 12 measures were identified. They included the peak flexion angle and sagittal-plane range of motion (ROM) at the LLS (LLSa_flexion_ and LLSa_range_) and at the ULS (ULSa_flexion_ and ULSa_range_); the peak angular velocity in flexion, the peak angular velocity in extension and the range between velocity peaks at the LLS (LLSv_flexion_, LLSv_extension_ and LLSv_range_) and at the ULS (ULSv_flexion_, ULSv_extension_ and ULSv_range_) and; the peaks of erector spinae muscle activity during the first (EMG_peak1_) and second (EMG_peak2_) halves of the tasks. Not all tasks presented the characteristic features necessary to the extraction of the 12 measures. Indeed, ULSv_flexion_, ULSv_extension_, ULSv_range_ and EMG_peak2_ were only present in flexion, picking-up and lifting. The measures were averaged over the repetitions in order to have only one value per participant and task. Finally, for EMG_peak1_ and EMG_peak2_, the maximal value observed between the left and right erector spinae muscles was kept for analysis.

**FIGURE 1:**
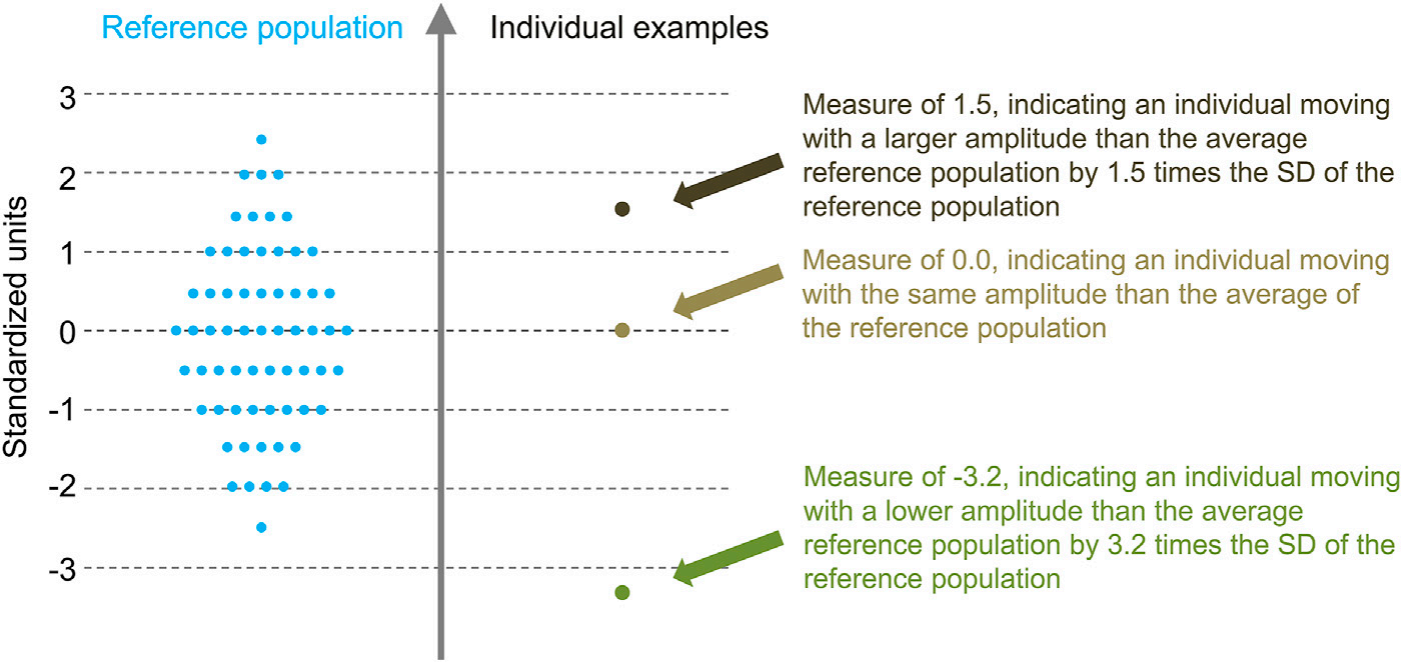
Illustration of the task-independent measures concept. Task-independent measures are expressed according to the mean and SD of the reference population. These dimensionless measures therefore indicate how they situate compared to the reference population.

Task–independent measures were calculated by averaging the measures obtained with the diverse tasks. The averaging was done independently for each participant and measure. To give similar weight to all the tasks, a Z-score transformation was applied to the measures before averaging over the tasks. The transformations were based on the means and standard deviations of the asymptomatic controls. Consequently, as illustrated in Figure 1, the task-independent measures were dimensionless: their values indicated how they were situated compared to the reference (asymptomatic) population. All calculations were performed with Matlab (R2019b, MathWorks, Inc, Natick, MA).

### Statistical Analysis

The normality of the measures was assessed visually using QQ plots and tested with the Shapiro-Wilk test (Ghasemi and Zahediasl, 2012). Extreme outliers were discarded from the analyses using a standard procedure (Portney and Watkins, 2000).

For the first aim, relationships among functional tasks in patients with CLBP were tested with Pearson correlations. These analyses were performed separately for each of the 12 measures. Correlation coefficients (*r*) were interpreted as small (0.1 ≤ *r <* 0.3), medium (0.3 ≤ *r <* 0.5) and large (*r ≥* 0.5) (Cohen, 1988).

For the second aim, we conducted binary logistic regression models with the movement measures as the independent variable and the group as the dependent variable. Regression models were performed separately for each measure of each task. The performance to differentiate patients with CLBP from asymptomatic controls was primarily tested with the area under the curve (AUC) value. These values were categorized as poor (AUC<0.7), acceptable (0.7 ≤ AUC <0.8), excellent (0.8 ≤ AUC <0.9) or outstanding (AUC≥ 0.9) discriminations (Hosmer et al., 2013). Following these categories, a difference in AUC between tasks above 0.1 was considered indicative of a relevant difference in groups’ differentiation performance. For completeness, other usual statistics of logistic regression models were calculated: coefficient of determination (*r* ^2^), sensitivity (Sn), specificity (Sp), positive likelihood ratios (LR+) and negative likelihood ratios (LR−). In addition, independent t-tests were conducted to determine if group differences were statistically significant and Cohen’s *d* effect sizes (ES) were computed to quantify the size of the differences between groups (Cohen, 1988). ES were interpreted as very small (0.01 ≤ ES < 0.2), small (0.2 ≤ ES < 0.5), moderate (0.5 ≤ ES < 0.8), large (0.8 ≤ ES < 1.2), very large (1.2 ≤ ES < 2.0) and huge (ES ≥ 2.0) (Cohen, 1988; Sawilowsky, 2009).

For the third aim regarding the construct validity of task–independent measures of spinal movement, binary logistic regression models were conducted for each of the task–independent measure. AUC values were interpreted as detailed above for the tasks’ comparison. Independent t-tests and ES were also calculated for task–independent measures. For completeness with the first aim, Pearson correlations were performed between task-independent and task-specific data for each measure. Statistical analyses were performed with SPSS (Version 25, IBM, NY, United States), using a significance level at α < 0.05.

### Sample Size

To detect a correlation coefficient between functional tasks of r ≥ 0.4, as reported in prior asymptomatic and pilot studies (Alqhtani et al., 2015; Christe et al., 2016a; Bujang and Baharum, 2016), with a power of 0.8 and α error of 0.05, the minimum sample size in the patients’ group was 46. For the logistic regression, the usual recommendations were followed, indicating a minimum of 15 participants per group in the case of models with a single independent variable (Portney and Watkins, 2000). Five asymptomatic participants and six participants with CLBP were added to prevent insufficient power due to potential drop-out or corrupted movement data.

## Results

Fifty-two patients with CLBP (sex: 63.5% male; age (mean ± SD): 40.0 ± 10.4 years old; BMI: 25.3 ± 3.3 kg/m^2^) and 20 asymptomatic controls (55% male; 38.2 ± 10.9 years old; 22.7 ± 2.8 kg/m^2^) were included in the study. The mean 24 h-NPRS, TSK, PCS and ODI scores of the patients were 5.6 ± 2.1, 44.3 ± 7.5, 25.2 ± 11.7 and 35.3 ± 11.2, respectively. Mean pain during movement was 4.6 ± 2.5 during flexion, 4.8 ± 2.7 during lifting, 4.3 ± 2.6 during picking-up, 2.7 ± 2.2 during stepping-up and 2.7 ± 2.5 during sit-to-stand for the patients. Mean perceived harm by the patients for each movement was 4.5 ± 3.5 for flexion, 6.3 ± 3.1 for lifting, 5.4 ± 3.2 for picking-up, 1.9 ± 2.4 for stepping-up and 2.6 ± 3.0 for sit-to-stand. Mean movement measures are reported in Supplementary Appendix SB for both groups. Movement data were available for at least 48 CLBP patients and 17 asymptomatic controls.

**FIGURE 2:**
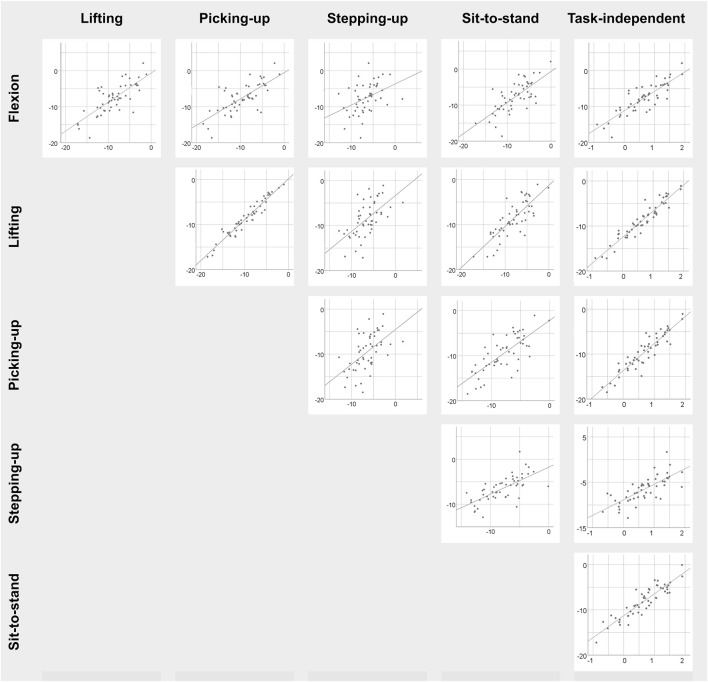
Scatterplots of LLSa_flexion_ among the five specific functional tasks as well as with respect to the task-independent LLSa_flexion_ measure in patients with CLBP. Task-specific measures are in degree and task-independent measures are dimensionless.

Correlations between tasks were statistically significant in 91 out of 99 (92%) cases (0.31 ≤ r ≤ 0.96, all *p* < 0.05) and 59 (60%) had a coefficient above 0.5 (Table 1; Figure 2). For angular amplitude measures, all but one correlation coefficients (39/40) were significant (*p* ≤ 0.01). All significant coefficients were above 0.3 and 28 (70%) exceeded 0.5. Correlation coefficients were significant and above 0.3 (*p* < 0.05) in 31/39 (79%) cases for angular velocity measures. The coefficients for angular velocity measures were larger than 0.5 in 16 (41%) cases. All correlations coefficients were significant and above 0.3 (*p* < 0.05) for muscle activity measures and 14/19 were above 0.5 (74%).

**TABLE 1:** Correlations in angular amplitude, angular velocity and muscle activity between different functional tasks. The darkness of the blue represents the strength of the correlation: white: r < 0.3; pale blue: 0.3 ≤ r < 0.5; blue: 0.5 ≤ r < 0.7; dark blue: r ≥ 0.7. Correlations in blue (r ≥ 0.3) all have a p-value <0.05. NA: variables not available as there were no characteristic pattern (see Data Processing). STS: sit-to-stand; LLS: Lower lumbar spine; ULS: Upper lumbar spine. EMG_peak1_: first peak of maximal paraspinal muscles activity; EMG_peak2_: second peak of maximal paraspinal muscles activity. Correlations between EMG_peak2_ of flexion, lifting and picking-up and EMG_peak1_ of stepping-up and sit-to-stand are reported in the last line (during stepping-up and sit-to-stand, there is only one peak of paraspinal muscle activity, see Data Processing).

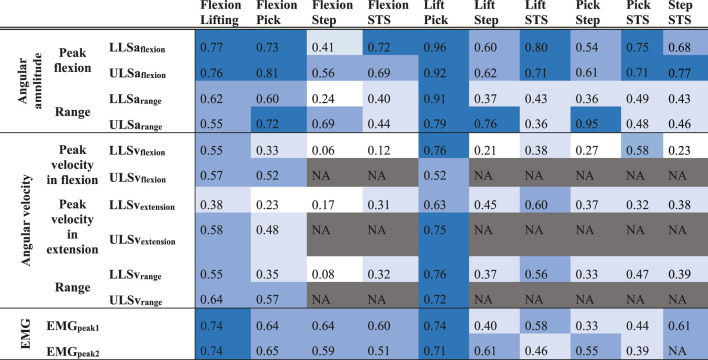

The AUC with their 95% confidence interval (CI), as well as the other performance values, of each movement measure in each functional task are presented in Tables 2–4. For the angular amplitude measures, five comparisons between tasks out of 40 (13%) reported a difference of AUC above 0.1 (range 0.11–0.15). Performance to differentiate groups during sit-to-stand compared to flexion and lifting was higher for ULSa_range_ (AUC of 0.74 compared to AUC of 0.63 and 0.59, respectively), but was lower for LLSa_range_ (AUC of 0.6 compared to AUC of 0.75 for flexion and AUC of 0.72 for lifting). AUC was also lower during lifting (AUC of 0.66) compared to flexion (AUC of 0.77) for LLSa_flexion_. Regarding angular velocity measures, 7 comparisons out of 42 (17%) reported a difference of AUC above 0.1 (range 0.11–0.14). For LLSv_flexion_, differentiation performance was smaller during sit-to-stand (AUC of 0.76) compared to picking-up, lifting and flexion (0.87 ≤ AUC ≤0.90), and smaller during stepping-up (AUC of 0.79) compared to picking-up (AUC of 0.90). AUC during picking-up for LLSv_extension_ (AUC of 0.88) was higher than during stepping-up (AUC of 0.75), and was also higher for LLSv_range_ (AUC of 0.92) compared to stepping-up (AUC of 0.79) and sit-to-stand (AUC of 0.81). Five out of 14 comparisons (36%) for muscle activity measures reported a difference in AUC above 0.1 (range 0.13–0.17). AUC during stepping-up (AUC of 0.51) was lower than during picking-up, lifting and flexion (0.64 ≤ AUC ≤0.68) for EMG_peak1_. For EMG_peak2_, flexion and lifting had higher AUC (AUC of 0.60 and 0.59, respectively) compared to picking-up (AUC of 0.47).

**TABLE 2:** Logistic regression models for angular amplitude measures. AUC: area under the curve [with its confidence interval (95%CI)]; *r* ^2^: coefficient of determination; Sn: sensitivity; Sp: specificity; LR+: positive likelihood ratio; LR−: negative likelihood ratio. Symbols (°^, §, #,^ *) indicate a difference of AUC >0.1 between two tasks or between a specific-task and the task–independent measure.

Variable	Task	AUC	95%CI			*r* ^2^	Sn	Sp	LR+	LR-
LLSa_flexion_	Flexion	0.77°	0.64	—	0.9	0.25	92.3	25	1.23	0.31
	Lifting	0.66°	0.5	—	0.81	0.1	100	5.3	1.06	0
	Picking-up	0.69	0.54	—	0.84	0.16	96.1	26.3	1.3	0.15
	Stepping-up	0.67	0.52	—	0.82	0.14	98	26.3	1.33	0.08
	Sit-to-stand	0.7	0.56	—	0.84	0.17	98.1	25	1.31	0.08
	Task–independent	0.71	0.58	—	0.85	0.18	94.2	35	1.45	0.17
LLSa_range_	Flexion	0.75^#^	0.62	—	0.89	0.26	94.2	40	1.57	0.15
	Lifting	0.72^§^	0.57	—	0.87	0.22	94.2	26.3	1.28	0.22
	Picking-up	0.68	0.54	—	0.82	0.17	96.1	21.1	1.22	0.18
	Stepping-up	0.65	0.51	—	0.8	0.11	96.1	15.8	1.14	0.25
	Sit-to-stand	0.60^# §^ *	0.45	—	0.75	0.05	100	10	1.11	0
	Task–independent	0.71*	0.57	—	0.86	0.22	98.1	30	1.4	0.06
ULSa_flexion_	Flexion	0.56	0.42	—	0.7	0.01	100	0	1	-
	Lifting	0.57	0.43	—	0.71	0.02	100	0	1	-
	Picking-up	0.59	0.44	—	0.73	0.03	100	0	1	-
	Stepping-up	0.59	0.45	—	0.74	0.05	100	5	1.05	0
	Sit-to-stand	0.61	0.46	—	0.75	0.05	100	5	1.05	0
	Task–independent	0.59	0.45	—	0.74	0.03	100	0	1	-
ULSa_range_	Flexion	0.63^#^	0.49	—	0.76	0.06	98.1	0	0.98	-
	Lifting	0.59^§^*	0.45	—	0.74	0.04	100	0	1	-
	Picking-up	0.69	0.56	—	0.83	1.77	96.2	30	1.37	0.13
	Stepping-up	0.67	0.54	—	0.8	0.12	96.2	20	1.2	0.19
	Sit-to-stand	0.74^# §^	0.61	—	0.87	0.22	96.2	30	1.11	0
	Task–independent	0.7*	0.57	—	0.83	0.16	96.2	20	1.2	0.2

**TABLE 3:** Logistic regression models for angular velocity measures. AUC: area under the curve [with its confidence interval (95%CI)]; *r* ^2^: coefficient of determination; Sn: sensitivity; Sp: specificity; LR+: positive likelihood ratio; LR−: negative likelihood ratio. Symbols (°^, §, #,^ *) indicate a difference of AUC >0.1 between two tasks or between a specific-task and the task–independent measure.

Variable	Task	AUC	95%CI			*r* ^2^	Sn	Sp	LR+	LR−
LLSv_flexion_	Flexion	0.89°	0.8	—	0.97	0.45	94.2	50	1.88	0.12
	Lifting	0.87^#^	0.79	—	0.96	0.45	92.3	52.6	1.95	0.15
	Picking-up	0.9^§^	0.82	—	0.98	0.59	94.1	63.2	2.56	0.09
	Stepping-up	0.79* ^§^	0.66	—	0.91	0.3	94.1	42.1	1.63	0.14
	Sit-to-stand	0.76° ^# §^ *	0.62	—	0.9	0.29	96.2	40	1.6	0.09
	Task–independent	0.9*	0.82	—	0.99	0.54	96.2	55	2.14	0.07
LLSv_extension_	Flexion	0.85	0.75	—	0.95	0.41	96.2	50	1.92	0.08
	Lifting	0.84	0.74	—	0.94	0.38	94.2	42.1	1.63	0.14
	Picking-up	0.88°	0.79	—	0.97	0.56	94.1	63.2	2.56	0.09
	Stepping-up	0.75° *	0.62	—	0.88	0.2	92	26.3	1.25	0.3
	Sit-to-stand	0.8	0.67	—	0.92	0.37	94.1	52.6	1.99	0.11
	Task–independent	0.88*	0.79	—	0.97	0.52	96.2	55	2.14	0.07
LLSv_range_	Flexion	0.88	0.78	—	0.97	0.48	96.2	55	2.14	0.07
	Lifting	0.89	0.82	—	0.97	0.49	92.3	52.6	1.95	0.15
	Picking-up	0.92°	0.85	—	0.99	0.62	96.1	57.9	2.28	0.07
	Stepping-up	0.79° *	0.66	—	0.92	0.31	92.2	36.8	1.46	0.21
	Sit-to-stand	0.81° ^§^	0.7	—	0.93	0.39	98.1	50	1.96	0.04
	Task–independent	0.92* ^§^	0.85	—	0.99	0.55	96.2	50	1.92	0.08
ULSv_flexion_	Flexion	0.88	0.8	—	0.96	0.51	92.3	55	2.05	0.14
	Lifting	0.93	0.85	—	1	0.67	96.2	78.9	4.56	0.05
	Picking-up	0.85	0.75	—	0.95	0.45	92.3	50	1.85	0.15
	Task–independent	0.94	0.88	—	1	0.69	94.2	65	2.69	0.1
ULSv_extension_	Flexion	0.83	0.73	—	0.92	0.3	92.3	40	1.54	0.19
	Lifting	0.82	0.71	—	0.92	0.27	92.3	17.6	1.12	0.44
	Picking-up	0.83	0.74	—	0.92	0.38	90.4	35	1.39	0.27
	Task–independent	0.88	0.8	—	0.95	0.45	88.5	55	1.97	0.2
ULSv_range_	Flexion	0.87	0.79	—	0.96	0.45	92.3	50	1.85	0.15
	Lifting	0.92	0.85	—	0.99	0.61	92.3	73.7	3.51	0.1
	Picking-up	0.88	0.8	—	0.96	0.51	92.3	65	2.64	0.12
	Task–independent	0.94	0.88	—	0.99	0.66	90.4	70	3.01	0.1

**TABLE 4:** Logistic regression models for lumbar muscle activity measures. AUC: area under the curve [with its confidence interval (95%CI)]; *r* ^2^: coefficient of determination; Sn: sensitivity; Sp: specificity; LR+: positive likelihood ratio; LR−: negative likelihood ratio. Symbols (°^, §, #,^ *) indicate a difference of AUC >0.1 between two tasks or between a specific-task and the task–independent measure.

Variable	Task	AUC	95%CI			*r* ^2^	Sn	Sp	LR+	LR-
EMG_peak1_	Flexion	0.68°	0.54	—	0.82	0.11	98.1	0	0.98	—
	Lifting	0.68^§^	0.5	—	0.82	0.1	98	0	0.98	-
	Picking-up	0.64^#^	0.5	—	0.78	0.09	100	0	1	—
	Stepping-up	0.51° ^§ #^ *	0.35	—	0.66	0.01	100	0	1	—
	Sit-to-stand	0.6	0.45	—	0.76	0.02	100	0	1	—
	Task–independent	0.64*	0.5	—	0.78	0.08	98.1	0	0.98	—
EMG_peak2_	Flexion	0.6°	0.44	—	0.75	0.05	98	5	1.03	0.4
	Lifting	0.59^§^	0.44	—	0.74	0.03	97.9	0	0.98	—
	Picking-up	0.47° ^§^	0.32	—	0.61	0	100	0	1	—
	Task–independent	0.55	0.41	—	0.7	0	100	0	1	—

**FIGURE 3:**
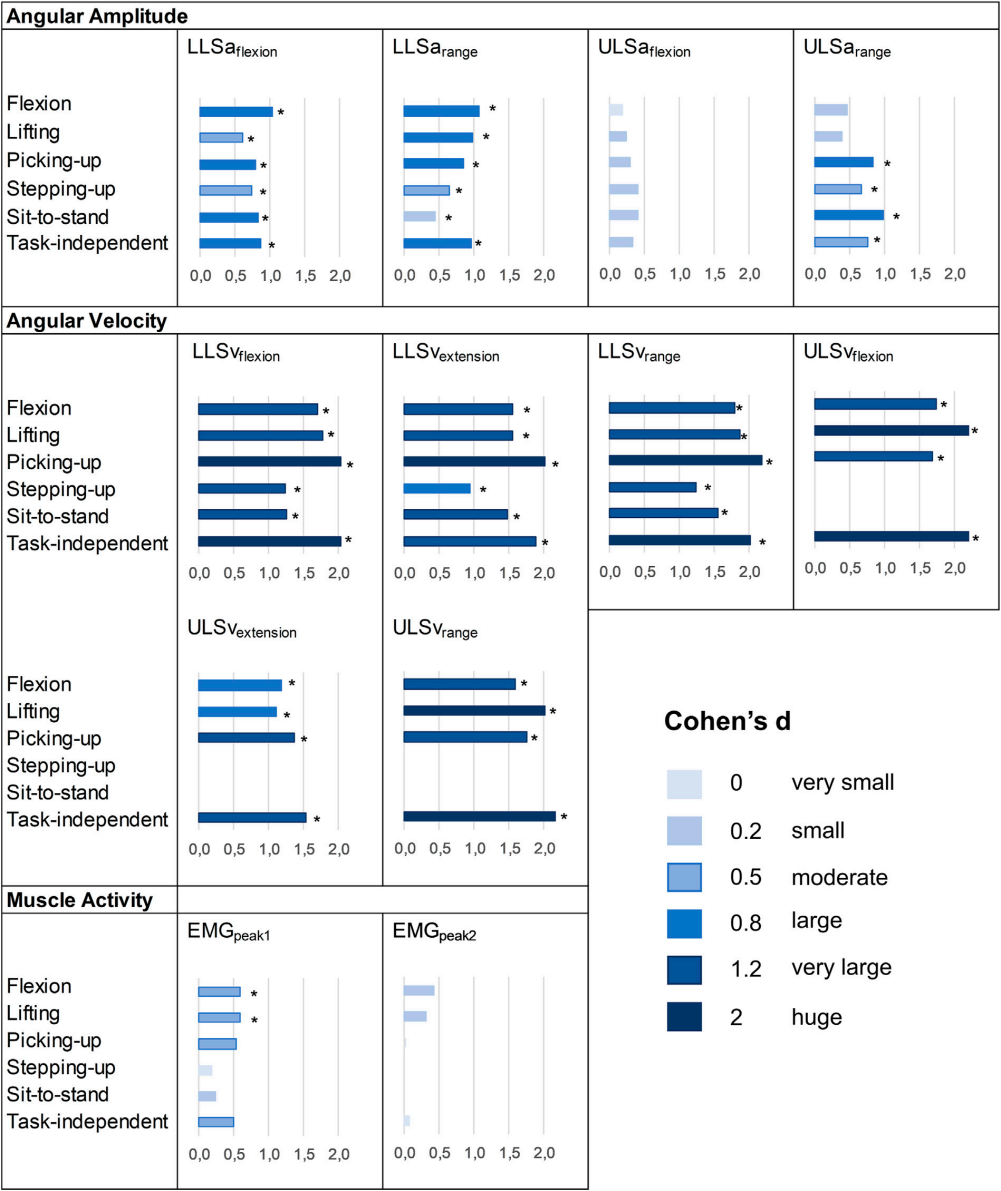
Effect sizes of the differences between patients with CLBP and asymptomatic controls. *: p-value<0.05 at t-tests. No ES means that the variable was not available because there was no characteristic pattern (see Data processing).

Results from independent t-tests and ES regarding the differences in movement measures between groups are reported in Figure 3 and Supplementary Appendix SB. Patients with CLBP moved with statistically significantly reduced LLSa_flexion_ and LLSa_range_ in all functional tasks compared to asymptomatic controls and all ES were moderate to large. At the ULS, ULSa_range_ during picking-up, stepping-up and sit-to-stand were significantly reduced in patients with CLBP, with moderate to large ES for the three tasks. Angular velocity measures were all significantly reduced in patients with CLBP, and ES were at least large. For muscle activity measures, EMG_peak1_ during flexion and lifting was significantly higher in patients with CLBP. ES ranged from 0.18 to 0.6 for EMG_peak1_ and from 0.02 to 0.42 for EMG_peak2_.

Task–independent measures reported AUC between 0.59 and 0.71, between 0.88 and 0.94 and between 0.55 and 0.64 for angular amplitude, angular velocity and muscle activity measures, respectively. Compared to each task, performance to differentiate groups for task–independent measures was superior (AUC difference >0.1) in 7/52 cases (13%) and was not different (AUC difference ≤0.1) in 45/52 cases (87%) (Tables 2–4). Correlation coefficients between task-independent and task-specific measures were large for angular amplitudes (range 0.63–0.95, all *p* < 0.001), angular velocities (range 0.74–0.92, all *p* < 0.001) and muscle activities (range 0.70–0.93, all *p* < 0.001) (Supplementary Appendix SC). Groups differences were statistically significant for LLSa_flexion_, LLSa_range_, ULSa_range_ and for all angular velocity measures. ES for angular amplitude measures ranged from 0.33 to 0.97, for angular velocity measures from 1.54 to 2.36 and for muscle activity measures from 0.07 to 0.51 (Figure 3 and Supplementary Appendix SB).

## Discussion

The correlation and capacity to differentiate patients from controls results indicated a limited influence of the tasks on the information carried by spinal movement measures and highlighted an individual spinal movement signature. This study also showed the construct validity of task–independent measures of spinal movement, and encouraged its consideration in future research. These important findings are discussed in the following sections.

### Consistency of Spinal Movement in Different Tasks

Ninety-two percent of the measures were significantly correlated between different functional tasks in patients with CLBP, with correlation coefficients demonstrating at least a medium effect (r ≥ 0.3). The coefficients were even large in 70, 41 and 76% of angular amplitude, angular velocity and muscle activity measures, respectively. Correlation coefficients tended to be larger for peak flexion angle at the lower (LLSa_flexion_) and upper lumbar spine (ULSa_flexion_), which is consistent with what was found in asymptomatic individuals (Alqhtani et al., 2015; Seerden et al., 2019). Interestingly, the present study also demonstrated individual consistency in lumbar angular velocity and in the level of muscle activity, suggesting that the consistency across tasks is not limited to angular amplitudes as previously shown (Alqhtani et al., 2015; Seerden et al., 2019). Correlation coefficients were very large between analogous functional tasks, such as lifting and picking-up, but consistency was also observed between differing tasks, such as flexion and sit-to-stand. While the large standard deviation in most measures of spinal movement in patients with CLBP showed heterogeneity between participants, the correlations between tasks demonstrated consistency within patients.

These findings support an individual spinal movement signature in the sagittal plane in patients with CLBP, suggesting that each individual has a consistent spinal movement across tasks. These results questioned the need to analyze multiple tasks involving primarily sagittal-plane movement independently and the need to investigate new primarily sagittal-plane functional tasks in future studies, as those may lead to redundant data, therefore complexifying the procedure without gaining information to improve our understanding of spinal movement in CLBP (Papi et al., 2018; Moissenet et al., 2021). These findings also suggest that spinal movement is probably more influenced by individual factors than by the tasks. However, which individual factors are associated with the spinal movement signature is still unclear. Previous research in patients with LBP reported association between spinal kinematics and pain intensity, psychological characteristics, sex, age and BMI, among others (Mitchell et al., 2008; Arshad et al., 2019; Christe et al., 2021). However, these factors demonstrated only small associations with spinal movement, suggesting that other unknown factors are in play. Consequently, further research is needed to better understand which factors influence the spinal movement signature in patients with CLBP.

### Difference in Performance to Differentiate Groups Between Functional Tasks

The functional tasks had little influence on the performance to differentiate patients with CLBP from asymptomatic controls. Less than 20% of the comparisons demonstrated a difference in AUC between tasks larger than 0.1. For angular amplitude measures, performance to differentiate patients from controls was consistent across the tasks in 79% of the comparisons. Independent t-tests, ES and other outcomes of models’ performance also supported these findings. When there was a difference, it was in the ranges of motion and not the peak amplitudes. However, differences were inconsistent among tasks, as sit-to-stand demonstrated higher performance at the ULSa_range_ and poorer performance at the LLSa_range_ compared to flexion and lifting. Regarding angular velocity measures, AUC did not differ substantially in 83% of the comparisons. When it differed, it consistently showed poorer performance during sit-to-stand and stepping-up. Although stepping-up and sit-to-stand also reported mostly very large effect sizes, their capacity to differentiate groups was smaller compared to flexion, lifting and picking-up. Regarding muscle activity measures, the capacity to differentiate groups was poor in all tasks and ES were moderate at most. The performance to differentiate groups was poorer during stepping-up for EMG_peak1_ and during picking-up for EMG_peak2_.

Globally, these observations indicate that assessing a range of functional tasks in the sagittal-plane may provide similar findings in terms of differentiating patients with CLBP from asymptomatic controls. The differences found between sit-to-stand and stepping-up compared to flexion, lifting and picking-up for angular velocity measures may be explained by the fact that participants with CLBP rated flexion, lifting and picking-up as more painful and more harmful for the back than stepping-up and sit-to-stand. The perceived harm might have led to increased pain-related fear and, together with the higher pain intensity, influenced angular velocity. Yet, these differences did not seem to consistently influence the angular amplitudes, questioning the effect of pain intensity and pain-related fear on lumbar angular amplitude. These findings are in line with a recent meta-analysis that showed a very small association between pain-related fear or pain intensity and spinal angular amplitudes, which was consistent across a wide range of tasks and measures of spinal angular amplitude (Christe et al., 2021). Therefore, although this study showed a redundancy among the tasks, it is possible that some functional tasks may be more sensitive, for example with respect to pain intensity or pain-related fear. In this regard, some authors suggested that selecting a specific task for each individual based on their identified limitations could be helpful when analyzing the relationships between spinal movement and patient-related outcomes (Wernli et al., 2020a). It is currently unknown if selecting one sagittal-plane task based on the individual limitation would be more appropriate to analyze such relationships and future studies should address this gap. If it would be the case, this would support the assessment of one specific primarily sagittal-plane task; the others tasks complexifying the procedure in vain (without brining supplementary information). If not, this might support the use of task–independent measures as discussed below.

### Task–Independent Measures of Spinal Movement

In this study, we showed the possibility and potential of averaging spinal movement measures across different functional tasks. Averaging spinal movement measures across multiple tasks was particularly supported by the individual spinal movement signature found in this study. The performance to differentiate groups was higher or did not differ compared to individual tasks in all the measures. Furthermore, angular velocity task–independent measures showed a high performance to differentiate groups, with statistically significant differences between the groups and very large to huge ES. ES were also large for the angular amplitude measures at the lower lumbar spine. Therefore, these results support the construct validity of task–independent measures of spinal movement and its consideration in future research.

Using an “average” measure across different tasks may have some interest in future studies. The method we used is simple as it consisted in averaging the Z-scores of each of the task, which could be easily replicated with any other biomechanical model or measure. These task–independent measures could notably be more robust because they are not reliant on a single task.

Nevertheless, future research is strongly recommended to determine the value of task–independent measures. Based on this study, it is not known if task–independent measures can provide more information than task-specific measures, nor how many movements should be averaged. Furthermore, reliability of averaging spinal movement measures from different functional tasks remains to be tested. While the method of averaging tasks using Z-scores has the advantage of its simplicity, other more advanced methods to group spinal movement measures (i.e., machine learning methods) will also need to be investigated.

### Capacity to Differentiate Patients With CLBP From Asymptomatic Controls

While it was not the objective of this study, the capacity of spinal movement measures to differentiate patients with CLBP from asymptomatic controls is worth discussing. First, patients with CLBP moved with reduced sagittal-plane lumbar amplitude and range of motion at the lower lumbar spine, in all functional tasks. ES were moderate to large. However, the low specificity and LR + suggested that small amplitudes are also frequent in asymptomatic controls. Second, angular velocity measures demonstrated very large ES in the majority of the tasks, which were always larger than the ES from angular amplitude or lumbar muscle activity measures. The capacity of angular velocity measures to differentiate patients from controls was even rated as outstanding for flexion, lifting and picking-up. The high sensitivity and very low LR− in all tasks showed that moving with high angular velocity was very rare in our sample of patients with CLBP, suggesting that moving at high angular velocity is very difficult with CLBP. Third, selected peaks of erector spinae activity demonstrated poor performance to differentiate groups. There were only two muscle activity measures that showed a statistically significant difference between the groups, and most ES were small. These findings are in agreement with previous studies analyzing erector spinae activity during dynamic tasks and reporting inconsistent results (Geisser et al., 2005). Yet, when a difference in muscle activity was observed between groups, it corresponded to higher levels of activity in patients with CLBP.

Overall, based on the present findings and previous reports (Shum et al., 2005; Laird et al., 2014; Christe et al., 2016b, 2020; Papi et al., 2018; Moissenet et al., 2021), reduced lumbar amplitude and angular velocity seems to be key characteristics of patients with CLBP. Furthermore, the consistent reduced sagittal-plane lumbar angular amplitude and velocity across all the functional tasks suggest that these spinal movement alterations generalize across a wide range of daily-life activities. Our results thus support the measurement of lumbar angular amplitude and angular velocity in any functional task in future studies. Nevertheless, there is an urgent need for well-conducted longitudinal studies to detangle if and how spinal kinematic changes are associated with patients’ changes in pain and disability (Wernli et al., 2020b; Schmid et al., 2021).

### Limitations

This study has some limitations that are important to discuss. First, the asymptomatic population was small, despite a number of participants above the minimum indicated by the sample size calculation (Portney and Watkins, 2000). While the number of participants had certainly little influence on the task comparisons, the performance values from the logistic regression models should be confirmed with larger groups. Second, the findings may not be transferable to all patients with CLBP. Although our results are consistent with current knowledge in the field, patients with CLBP included in this study had high levels of disability, pain-related fear and catastrophizing that are common in patients participating to interdisciplinary rehabilitation programs. These individual factors may have influenced the large differences found between the groups. Third, the tasks were not assessed in random order. Therefore, the higher level of pain found during picking-up and lifting may be related to the fact that these two tasks were collected last (order effect). Fourth, the video recordings used to present the daily-life tasks may have influenced how participants performed the tasks, requesting caution when interpreting the findings in the context of movement behavior. Video recordings were used to give standardized instructions and avoid differences in the ways of completing the tasks. This was particularly important for picking-up and lifting, as these tasks can be performed in different ways (i.e., stoop or squat). While the instructions could have limited inter-individual variability, large variations were observed among individuals, suggesting that participants were not too constrained and could express their individual movement signature. On the other hand, one cannot exclude that more pronounced signatures could have been observed if the tasks would have been less standardized. Finally, this study focused on sagittal-plane lumbar biomechanics during functional tasks primarily involving sagittal-plane movements, because this corresponds to the alterations the most frequently reported in literature and because patients often complain of movement-related pain in primarily sagittal-plane activities. Therefore, it has yet to be determined if other functional tasks with larger solicitations in the frontal and/or transverse–planes, such as gait (Christe et al., 2017; Schmid et al., 2017), would display individual movement signatures in these other planes or even three-dimensionally.

## Conclusion

This study showed that individuals with CLBP have consistent spinal movement in the sagittal plane across different functional tasks, supporting the existence of an individual biomechanical signature. Furthermore, the capacity to differentiate patients with CLBP from asymptomatic controls did not differ between functional tasks in most of the cases. Therefore, this study highlighted a redundancy among tasks, questioning the most appropriate measures to describe spinal movement behavior in the framework of CLBP. While further research will be necessary in this regard, this study showed the feasibility of task-independent measures, a promising approach towards an effective quantification of spinal movement.

## Data Availability

The raw data supporting the conclusion of this article will be made available by the authors, without undue reservation.
