# Supplementary material for: Patients With Chronic Low Back Pain Have an Individual Movement Signature: A Comparison of Angular Amplitude, Angular Velocity and Muscle Activity Across Multiple Functional Tasks
Source: Front Bioeng Biotechnol. 2021 Nov 15;9:767974. doi: 10.3389/fbioe.2021.767974 (PMC8634715; doi:10.3389/fbioe.2021.767974)
Supplement: Supplementary file 1 [file DataSheet1.docx]

Supplementary Material

**Appendix A**

Standardized protocol and instructions for the functional task. Before each of the tasks, a video recording presenting the task was shown to the participants.

1. Flexion

Instructions: “*Position your feet hip-width apart, keep your knees straight and bend forward as low as possible at your normal speed. Start by rolling up your head. At the bottom, stay for 3 seconds, then come up at your normal speed*.”

1. Sit-to-stand

The height of the stool is adjusted according to individual’s height, in order to have their thighs horizontal. Participants’ feet were positioned shoulder width apart, their arms relaxed and they had to take a normal upright sitting posture. Instructions: “*Stand up from the chair at your normal speed*”

1. Stepping-up

The distance between the step and the feet is 20 cm. A tape was placed on the floor to ensure that the starting position was the same across trials. Instructions: “*Step-up on the box with your right foot first at your normal speed*”.

1. Picking-up

The distance between the feet of the subjects and the sponge was standardized. The sponge was placed 15cm from the feet (centrally). Instructions: “*Pick-up the sponge from the floor at your normal speed with your right hand”.*

1. Lifting

The distance between the feet and the box was standardized and placed 4cm from the feet. Instructions: “*Lift the box from the floor at your normal speed with both hands”.*

**Appendix B**

Mean and standard deviation (SD) of the measures of interest for the asymptomatic participants (ASY) and the patients with CLBP (CLBP). The p-values and effect sizes (ES) in the latest column correspond to independent t-tests and Cohen’s *d* ES between both groups. This table also reports the number of participants (n) with measures available for the statistical analyses.

|  |  |  | **ASY** | |  | **LBP** | |  |  |
| --- | --- | --- | --- | --- | --- | --- | --- | --- | --- |
|  | **Measures** | **n** | **Mean** | **SD** | **n** | **Mean** | **SD** | **p-value** | **ES** |
| **Flexion** | ***Spinal amplitude*** |  |  |  |  |  |  |  |  |
|  | LLSa_flexion_ | 20 | -12,2 | 5,1 | 52 | -7,7 | 4,2 | <0.001 | 1,0 |
|  | LLSa_range_ | 20 | 14,6 | 5,2 | 52 | 10,0 | 3,9 | <0.001 | 1,1 |
|  | ULSa_flexion_ | 20 | -27,6 | 6,1 | 52 | -26,4 | 7,3 | 0.48 | 0,2 |
|  | ULSa_range_ | 20 | 29,3 | 5,0 | 52 | 26,3 | 6,9 | 0.09 | 0,5 |
|  | ***Angular velocity*** |  |  |  |  |  |  |  |  |
|  | LLSv_flexion_ | 20 | -14,2 | 6,9 | 52 | -6,1 | 4,0 | <0.001 | 1,7 |
|  | LLSv_extension_ | 20 | 13,3 | 6,6 | 52 | 6,8 | 3,4 | <0.001 | 1,5 |
|  | LLSv_range_ | 20 | 27,5 | 12,2 | 52 | 12,8 | 6,8 | <0.001 | 1,8 |
|  | ULSv_flexion_ | 20 | -29,5 | 9,6 | 52 | -16,4 | 6,8 | <0.001 | 1,7 |
|  | ULSv_extension_ | 20 | 25,0 | 6,5 | 52 | 16,4 | 7,5 | <0.001 | 1,2 |
|  | ULSv_range_ | 20 | 54,5 | 14,0 | 52 | 32,8 | 13,5 | <0.001 | 1,6 |
|  | ***Muscle activity*** |  |  |  |  |  |  |  |  |
|  | EMG_peak1_ | 20 | 0,3 | 0,1 | 52 | 0,4 | 0,2 | 0.03 | 0,6 |
|  | EMG_peak2_ | 20 | 0,8 | 0,3 | 52 | 0,7 | 0,3 | 0.12 | 0,4 |
|  |  |  |  |  |  |  |  |  |  |
| **Picking-up** | ***Spinal amplitude*** |  |  |  |  |  |  |  |  |
|  | LLSa_flexion_ | 19 | -13,2 | 5,7 | 51 | -9,6 | 4,1 | 0,004 | 0,8 |
|  | LLSa_range_ | 19 | 15,2 | 5,7 | 51 | 11,5 | 3,9 | 0,003 | 0,8 |
|  | ULSa_flexion_ | 20 | -24,1 | 7,1 | 52 | -22,0 | 7,1 | 0,26 | 0,3 |
|  | ULSa_range_ | 20 | 26,5 | 6,2 | 52 | 21,9 | 5,4 | 0,002 | 0,8 |
|  | ***Angular velocity*** |  |  |  |  |  |  |  |  |
|  | LLSv_flexion_ | 19 | -33,8 | 15,9 | 51 | -16,0 | 6,145 | <0.001 | 2,0 |
|  | LLSv_extension_ | 19 | 25,5 | 10,3 | 51 | 13,2 | 4,6 | <0.001 | 2,0 |
|  | LLSv_range_ | 19 | 59,2 | 24,7 | 51 | 29,2 | 9,8 | <0.001 | 2,2 |
|  | ULSv_flexion_ | 20 | -61,7 | 26,9 | 52 | -34,1 | 12,6 | <0.001 | 1,7 |
|  | ULSv_extension_ | 20 | 40,6 | 12,7 | 52 | 25,1 | 10,9 | <0.001 | 1,4 |
|  | ULSv_range_ | 20 | 102,3 | 34,0 | 52 | 59,2 | 21,3 | <0.001 | 1,7 |
|  | ***Muscle activity*** |  |  |  |  |  |  |  |  |
|  | EMG_peak1_ | 20 | 0,4 | 0,2 | 52 | 0,6 | 0,4 | 0,05 | 0,5 |
|  | EMG_peak2_ | 20 | 0,7 | 0,3 | 49 | 0,7 | 0,4 | 0,95 | 0,0 |
|  |  |  |  |  |  |  |  |  |  |

|  |  |  | **ASY** | |  | **LBP** | |  |  |
| --- | --- | --- | --- | --- | --- | --- | --- | --- | --- |
|  | **Measures** |  | **Mean** | **SD** |  | **Mean** | **SD** | **p-value** | **ES** |
| **Lifting** | ***Spinal amplitude*** |  |  |  |  |  |  |  |  |
|  | LLSa_flexion_ | 19 | -11,4 | 5,4 | 52 | -8,7 | 4,0 | 0,03 | 0,6 |
|  | LLSa_range_ | 19 | 14,8 | 5,2 | 52 | 10,9 | 3,5 | 0,006 | 1,0 |
|  | ULSa_flexion_ | 19 | -22,8 | 6,9 | 52 | -20,9 | 7,7 | 0,36 | 0,2 |
|  | ULSa_range_ | 19 | 23,2 | 5,0 | 52 | 21,0 | 6,2 | 0,16 | 0,4 |
|  | ***Angular velocity*** |  |  |  |  |  |  |  |  |
|  | LLSv_flexion_ | 19 | -31,0 | 13,9 | 52 | -14,5 | 7,6 | <0.001 | 1,8 |
|  | LLSv_extension_ | 19 | 22,6 | 10,3 | 52 | 12,1 | 5,5 | <0.001 | 1,6 |
|  | LLSv_range_ | 19 | 53,6 | 21,6 | 52 | 26,6 | 11,9 | <0.001 | 1,9 |
|  | ULSv_flexion_ | 19 | -50,9 | 12,9 | 52 | -28,3 | 9,1 | <0.001 | 2,2 |
|  | ULSv_extension_ | 17 | 31,8 | 6,5 | 52 | 21,7 | 10,0 | <0.001 | 1,1 |
|  | ULSv_range_ | 19 | 86,3 | 19,0 | 52 | 50,0 | 17,8 | <0.001 | 2,0 |
|  | ***Muscle activity*** |  |  |  |  |  |  |  |  |
|  | EMG_peak1_ | 18 | 0,5 | 0,2 | 51 | 0,7 | 0,3 | 0,04 | 0,6 |
|  | EMG_peak2_ | 19 | 0,9 | 0,4 | 48 | 0,8 | 0,4 | 0,26 | 0,3 |
|  |  |  |  |  |  |  |  |  |  |
| **Stepping-up** | ***Spinal amplitude*** |  |  |  |  |  |  |  |  |
|  | LLSa_flexion_ | 19 | -8,9 | 4,1 | 51 | -6,5 | 2,9 | 0,008 | 0,7 |
|  | LLSa_range_ | 19 | 10,8 | 3,7 | 51 | 8,7 | 3,0 | 0,02 | 0,6 |
|  | ULSa_flexion_ | 20 | -11,9 | 5,7 | 52 | -9,7 | 5,3 | 0,12 | 0,4 |
|  | ULSa_range_ | 20 | 24,3 | 5,3 | 52 | 20,5 | 5,7 | 0,01 | 0,7 |
|  | ***Angular velocity*** |  |  |  |  |  |  |  |  |
|  | LLSv_flexion_ | 19 | -26,2 | 12,1 | 51 | -16,0 | 6,9 | 0,002 | 1,2 |
|  | LLSv_extension_ | 19 | 21,3 | 8,9 | 50 | 14,6 | 6,4 | 0,001 | 0,9 |
|  | LLSv_range_ | 19 | 47,5 | 16,4 | 51 | 31,0 | 12,2 | <0.001 | 1,2 |
|  | ***Muscle activity*** |  |  |  |  |  |  |  |  |
|  | EMG_peak_ | 20 | 0,8 | 0,4 | 50 | 0,7 | 0,3 | 0,5 | 0,2 |
|  |  |  |  |  |  |  |  |  |  |

|  |  |  | **ASY** | |  | **LBP** | |  |  |
| --- | --- | --- | --- | --- | --- | --- | --- | --- | --- |
|  | **Measures** |  | **Mean** | **SD** |  | **Mean** | **SD** | **p-value** | **ES** |
| **Sit-to-stand** | ***Spinal amplitude*** |  | **Mean** | **SD** |  | **Mean** | **SD** |  |  |
|  | LLSa_flexion_ | 20 | -11,4 | 5,2 | 52 | -8,1 | 3,5 | 0,02 | 0,8 |
|  | LLSa_range_ | 20 | 11,1 | 4,2 | 52 | 9,7 | 2,8 | 0,01 | 0,4 |
|  | ULSa_flexion_ | 20 | -21,1 | 9,5 | 52 | -17,7 | 7,8 | 0,13 | 0,4 |
|  | ULSa_range_ | 20 | 23,4 | 9,9 | 52 | 15,6 | 7,2 | <0.001 | 1,0 |
|  | ***Angular velocity*** |  |  |  |  |  |  |  |  |
|  | LLSv_flexion_ | 20 | -22,45 | 16,6 | 52 | -10,9 | 6,478 | 0,007 | 1,3 |
|  | LLSv_extension_ | 19 | 30,3 | 9,4 | 51 | 10,9 | 5,2 | <0.001 | 1,5 |
|  | LLSv_range_ | 20 | 45,6 | 23,8 | 52 | 22,5 | 11,6 | <0.001 | 1,6 |
|  | ***Muscle activity*** |  |  |  |  |  |  |  |  |
|  | EMG_peak_ | 20 | 0,8 | 0,3 | 50 | 0,8 | 0,3 | 0,39 | 0,2 |
|  |  |  |  |  |  |  |  |  |  |
| **Task-independent** | ***Spinal amplitude*** |  |  |  |  |  |  |  |  |
|  | LLSa_flexion_ | 20 | 0,02 | 0,94 | 52 | 0,65 | 0,65 | 0,002 | 0,9 |
|  | LLSa_range_ | 20 | 0,00 | 0,88 | 52 | -0,62 | 0,54 | 0,007 | 1,0 |
|  | ULSa_flexion_ | 20 | 0,02 | 0,87 | 52 | 0,30 | 0,89 | 0,22 | 0,3 |
|  | ULSa_range_ | 20 | 0,44 | 0,84 | 52 | -0,25 | 0,95 | 0,005 | 0,8 |
|  | ***Angular velocity*** |  |  |  |  |  |  |  |  |
|  | LLSv_flexion_ | 20 | -0,01 | 0,80 | 52 | 0,98 | 0,37 | <0.001 | 2,0 |
|  | LLSv_extension_ | 20 | -0,02 | 0,77 | 52 | -0,94 | 0,39 | <0.001 | 1,9 |
|  | LLSv_range_ | 20 | -0,31 | 0,72 | 52 | -1,30 | 0,41 | <0.001 | 2,0 |
|  | ULSv_flexion_ | 20 | 0,00 | 0,75 | 52 | 1,38 | 0,53 | <0.001 | 2,4 |
|  | ULSv_extension_ | 20 | -0,06 | 0,61 | 52 | -1,21 | 0,79 | <0.001 | 1,5 |
|  | ULSv_range_ | 20 | 0,00 | 0,72 | 52 | -1,57 | 0,74 | <0.001 | 2,2 |
|  | ***Muscle activity*** |  |  |  |  |  |  |  |  |
|  | EMG_peak1_ | 20 | 0,01 | 0,83 |  | 0,56 | 1,19 | 0,06 | 0,5 |
|  | EMG_peak2_ | 20 | -0,01 | 0,95 | 51 | -0,09 | 1,21 | 0,8 | 0,1 |

**Appendix C**

Correlation coefficients (r) in angular amplitude, angular velocity and muscle activity between task-independent measures and task-specific measures. NA: variables not available as there were no characteristic pattern (see Data processing). LLS: Lower lumbar spine; ULS: Upper lumbar spine. EMGpeak1: first peak of maximal paraspinal EMG activity; EMGpeak2: second peak of maximal paraspinal EMG activity. All correlation coefficients have a p-value<0.001.

|  |  |  | **r between the task-independent measures and the task-specific measures obtained during:** | | | | |
| --- | --- | --- | --- | --- | --- | --- | --- |
|  |  |  | **Flexion** | **Lifting** | **Picking-up** | **Stepping-up** | **Sit-to-stand** |
| **Angle** | **Peak flexion** | **LLS** | 0,89 | 0,94 | 0,94 | 0,83 | 0,92 |
|  |  | **ULS** | 0,89 | 0,91 | 0,93 | 0,79 | 0,84 |
|  | **Range** | **LLS** | 0,82 | 0,91 | 0,91 | 0,75 | 0,78 |
|  |  | **ULS** | 0,83 | 0,82 | 0,95 | 0,93 | 0,63 |
| **Angular velocity** | **Peak velocity in flexion** | **LLS** | 0,84 | 0,91 | 0,92 | 0,76 | 0,76 |
|  |  | **ULS** | 0,88 | 0,91 | 0,85 | 0,74 | 0,78 |
|  | **Peak velocity in extension** | **LLS** | 0,84 | 0,90 | 0,89 | 0,74 | 0,85 |
|  |  | **ULS** | 0,88 | 0,81 | 0,88 | NA | NA |
|  | **Range** | **LLS** | 0,88 | 0,92 | 0,92 | NA | NA |
|  |  | **ULS** | 0,90 | 0,90 | 0,88 | NA | NA |
| **EMG** | **EMGpeak1** | | 0,91 | 0,88 | 0,81 | 0,70 | 0,75 |
|  | **EMGpeak2** | | 0,88 | 0,93 | 0,93 | NA | NA |
